# SNP-markers in *Allium* species to facilitate introgression breeding in onion

**DOI:** 10.1186/s12870-016-0879-0

**Published:** 2016-08-31

**Authors:** Olga E. Scholten, Martijn P.W. van Kaauwen, Arwa Shahin, Patrick M. Hendrickx, L.C. Paul Keizer, Karin Burger, Adriaan W. van Heusden, C. Gerard van der Linden, Ben Vosman

**Affiliations:** Wageningen UR Plant Breeding, Wageningen University and Research Centre, PO Box 386, Wageningen, 6700 AJ The Netherlands

**Keywords:** *Allium cepa*, *A. roylei*, *A. fistulosum*, Interspecific hybrids, Transcriptome sequencing, *Botrytis squamosa*

## Abstract

**Background:**

Within onion, *Allium cepa* L., the availability of disease resistance is limited. The identification of sources of resistance in related species, such as *Allium roylei* and *Allium fistulosum*, was a first step towards the improvement of onion cultivars by breeding. SNP markers linked to resistance and polymorphic between these related species and onion cultivars are a valuable tool to efficiently introgress disease resistance genes. In this paper we describe the identification and validation of SNP markers valuable for onion breeding.

**Results:**

Transcriptome sequencing resulted in 192 million RNA seq reads from the interspecific F1 hybrid between *A. roylei* and *A. fistulosum* (RF) and nine onion cultivars. After assembly, reliable SNPs were discovered in about 36 % of the contigs. For genotyping of the interspecific three-way cross population, derived from a cross between an onion cultivar and the RF (CCxRF), 1100 SNPs that are polymorphic in RF and monomorphic in the onion cultivars (RF SNPs) were selected for the development of KASP assays. A molecular linkage map based on 667 RF-SNP markers was constructed for CCxRF. In addition, KASP assays were developed for 1600 onion-SNPs (SNPs polymorphic among onion cultivars). A second linkage map was constructed for an F2 of onion x *A. roylei* (F2(CxR)) that consisted of 182 onion-SNPs and 119 RF-SNPs, and 76 previously mapped markers. Markers co-segregating in both the F2(CxR) and the CCxRF population were used to assign the linkage groups of RF to onion chromosomes. To validate usefulness of these SNP markers, QTL mapping was applied in the CCxRF population that segregates for resistance to *Botrytis squamosa* and resulted in a QTL for resistance on chromosome 6 of *A. roylei*.

**Conclusions:**

Our research has more than doubled the publicly available marker sequences of expressed onion genes and two onion-related species. It resulted in a detailed genetic map for the interspecific CCxRF population. This is the first paper that reports the detection of a QTL for resistance to *B. squamosa* in *A. roylei*.

**Electronic supplementary material:**

The online version of this article (doi:10.1186/s12870-016-0879-0) contains supplementary material, which is available to authorized users.

## Background

Onion, *Allium cepa* L., is an important vegetable crop that is grown worldwide [1]. The economic importance of the crop has led to many different cultivars for various latitudes as bulb formation and therefore yield is highly dependent on day length. Yield is the major trait of any onion-breeding programme. To achieve maximum yield, increased levels of resistance to pests and diseases are needed to prevent yield losses. Through selection within *A. cepa* partial resistance or field resistance was obtained to fusarium basal rot and pink root in onion cultivars [2]. As resistance to pests and diseases is often not present in the crop species itself, introduction of these traits from crossable wild relatives can be a solution [3, 4]. For onion, downy mildew resistance is an example of a trait that was identified in *A. roylei* Stearn [5] and successfully introgressed in onion cultivars [6]. In *A. roylei* resistance to *Botrytis squamosa* [7, 8], and *Colletotrichum gloeosporioides* [9] was discovered as well. Onion lines with resistance to *B. squamosa* from *A. roylei* are currently being developed [10]. In another relative of onion, *A. fistulosum*, accessions with resistance to *B. squamosa* [8, 11, 12], *Fusarium oxysporum* [13], *Phoma terrestris* [14, 15] and *C. gloeosporioides* [9] were identified. Resistance to *F. oxysporum* was also observed in accessions of *A. galanthum* and *A. schoenoprasum* [16]. These examples clearly show the potential of onion-related species as sources for improvement of onion cultivars.

The use of crop wild relatives as a source of interesting genes/alleles in breeding is often complicated by amongst others crossing barriers, which may cause low or intermediate levels of fertility in progeny plants [17] or low levels of recombination in certain genomic regions. For instance, there are crossing barriers between onion and *A. fistulosum* resulting in sterility of F1 plants, even though they have the same number of chromosomes (2n = 2x = 16). The genome size of these species is different with 2C values of 33.5 and 22.5 pg for onion and *A. fistulosum*, respectively [18]. Although the genome size of *A. roylei* also differs from onion (2C = 28.5 pg), fertile progeny plants were obtained after crossing this species with onion [19], allowing the creation of a genetic linkage map using an F2 population [20, 21]. *Allium roylei* was also used as a bridge between onion and *A. fistulosum*, resulting in the production of a fertile interspecific three-way cross *A. cepa* x (*A. roylei* x *A. fistulosum*) population [22]. In these plants recombination events occurred between all chromosomes of *A. fistulosum* and *A. roylei,* as well as between the chromosomes of the F1 and onion after back crossing the three-way crossed plants with onion [23]. The occurrence of recombination demonstrated the potential for introgressing traits of *A. fistulosum* into *A. cepa* via *A. roylei*.

The development of molecular markers greatly facilitates the introgression of traits or genes from related species. Currently, single nucleotide polymorphisms (SNPs) are the markers of choice and large sets of SNPs have been developed between two inbred lines of onion (OH1 and 5225) [24] and also for *A. fistulosum* [25]. This paper describes the development of SNP markers between two species of *Allium* (*A. roylei* and *A. fistulosum*) using the interspecific F1 hybrid (called RF) and a set of onion cultivars (CC). As the *Allium* genome is large (16 Gbp = 18× tomato) [26] transcriptome sequencing was used to reduce complexity and to increase the chance of tagging single copy regions with high enough redundancy to reliably discover useful SNPs. RF-SNPs heterozygous in RF and homozygous in onion cultivars were used to create a molecular linkage map using the *A. cepa* x (*A. roylei* x *A. fistulosum*) interspecific three-way cross population [22, 27] (further called CCxRF). RF-SNPs that were polymorphic between *A. roylei* and onion were also mapped in the F2 population resulting from a cross between onion and *A. roylei*, named F2(CxR). In addition SNPs were discovered in onion cultivars. SNPs polymorphic between onion and *A. roylei* were also mapped on the CxR map to obtain a combined map for both the onion SNPs and the RF-SNPs, which is likely to improve the utilisation of the RF markers in onion breeding. The application and usefulness of the marker dataset and genetic map in breeding is exemplified by the identification of a QTL for resistance to *Botrytis* leaf blight (BLB) using the CCxRF population.

## Methods

### Plant materials

For SNP discovery, a vegetatively propagated interspecific hybrid plant RF (PRI 91021–8), originating from a cross between *A. roylei* and *A. fistulosum* [22] and nine onion cultivars (one plant per cultivar) were used originating from different origins in the world and differing in day length dependency (Table 1). Two populations were used for mapping, the interspecific three-way cross population CCxRF and the F2 population F2(CxR). The three-way cross population was obtained after crossing male-sterile onion plants (an ‘A line’ or cultivar ‘Hygro’) with RF in successive years. Progeny plants of these crosses were used for resistance screening, as they were kept in tissue culture for several years. In 2011, a cross between one plant of ‘Hygro’ and the RF plant resulted in 154 progeny plants, that were used for mapping. The F2 population was obtained by selfing one plant of F1(CxR) (PRI 93103), a hybrid between *A. cepa* and *A. roylei* and consisted of 67 genotypes previously used for mapping [20, 21] and 32 newly added F2 plants.

**Table 1:** Onion accessions and cultivars, types and origins, used for SNP discovery

Genotype	Type^a^	Origin^b^
RF	NA	PRI 91021-8, Wageningen UR Plant Breeding, The Netherlands
Bravo	LLD	Rijnsburger, Hazera Seeds, The Netherlands
Jumbo	LLD	Rijnsburger, Syngenta, The Netherlands
Babosa	SD	Early Grano, CGN 15746, originally from Spain, imported in USA in 1925
California Red	SD	HRIGRU 5548, USA
Pukekohe Longkeeper	ID	HRIGRU 5524, Australia
Rio Tinto	SD	Bayer CropScience Vegetable Seed
Rumba	ID	Bayer CropScience Vegetable Seeds
Sapporo Yellow Globe	LD	CGN 14724, Japan
South Port White Globe	LD	CGN 14735, USA, 1906

^a^ *NA* not applicable, *LLD* Long long day, *LD* long day, *ID* intermediate day, *SD* short day

^b^ CGN is the Centre for Genetic Resources, the Netherlands, (www.cgn.wur.nl), HRIGRU is the Warwick Genetic Resources Unit, United Kingdom (www2.warwick.ac.uk/fac/sci/lifesci/wcc/gru/)

### Transcriptome sequencing, data processing and assembly

For SNP discovery, RNA was isolated from leaves of 4–8 week old plants (one plant per cultivar, grown in the greenhouse) using the Trizol protocol (Invitrogen, Carlsbad, Ca, USA) and purified using the RNeasy MinElute kit (Qiagen, Hilden, Germany). cDNA synthesis and bar-coding per sample, followed by paired-end Illumina sequencing were carried out by BaseClear (www.baseclear.com). The Illumina Genome analyzer HiSeq2000 was used to sequence three cDNA samples in a first run (RF and cultivars Bravo and Jumbo) and in the second run two cDNA samples of eight cultivars were multiplexed per lane (Table 1). Paired end reads from fragments shorter than 2× the read length were merged to one single elongated read using the stitch software (github.com/audy/stitch). The resulting sequence fragments and the unjoined reads were all imported as single reads in the CLC Genomics Workbench (v 4.03). Before assembly the reads were processed to increase quality by removal of: a) the first 5′ base, b) sequences with more than one ambiguous nucleotide (N), c) low quality bases on both ends of the reads. *De novo* assembly with the software CLC was carried out using the following settings: a) 95 % sequence homology over minimal 80 % of the read length; b) mismatch/insertion/deletion costs were set to 2,3,3, respectively; c) global alignment; d) the consensus sequence per contig for variable positions is determined by the most abundant nucleotide; e) reads that match more than one contig (non-specific matches) are not incorporated into any contig, and; f) a minimum contig length of 180 bp is required. Contigs were constructed for each genotype separately and for the onion genotypes combined.

### SNP discovery

All contigs were submitted to QualitySNP^ng^ for SNP discovery using the default settings of the programme [28, 29]. QualitySNP^ng^ is designed for filtering out SNPs from paralogous sequences without prior knowledge about the reference sequence. If the number of haplotypes exceeded two per SNP locus this is most likely the result of either paralogs assembled in one contig or sequencing artefacts. SNP regions were selected to have 75 bp flanking the SNP position at both sides without additional polymorphisms in these flanking regions. SNP regions were compared with all contigs using BlastN with Expectation value E lower than −20 to select unique SNP regions. Only SNP regions that mapped uniquely were kept [30]. These SNP markers were considered as usable.

### SNP selection and genotyping

Two types of SNP markers were selected for genotyping and construction of molecular linkage maps: RF-SNP markers (names start with RF_ctg ) and onion-SNP markers (names start with al_ctg). Selection criterion used for RF-SNP markers were that SNPs were polymorphic in the RF-hybrid and monomorphic in all tested onion cultivars. For onion-SNP markers, the criterion was presence of the major allele in at least five cultivars and the minor allele in at least four cultivars. Genotyping was done with the use of KASP™ assay by VHL Genetics (www.vhlgenetics.com) for the RF-SNPs, and by LGC Genomics (www.lgcgroup.com) for the onion-SNPs.

For genotyping, DNA was extracted [31] from young leaves of the CCxRF population, the F2(CxR) population and parental plants of these populations. For the onion parent, plants of onion cultivars ‘Jumbo’ and ‘Bravo’ were used. Parents of the populations were genotyped at least in triplicate to test reproducibility of the KASP assay. SNP data were visualised using the software programme SNPViewer of LGC Genomics (www.lgcgroup.com/products/genotyping-software/snpviewer).

### Genetic mapping

For the CCxRF population, only the SNPs polymorphic between the *A. roylei* parent and the *A. fistulosum* parent were used. These markers are expected to segregate in a 1:1 ratio as the selected SNP markers were polymorphic in the RF hybrid (heterozygous) and monomorphic in onion (selected for homozygosity of the onion allele). A genetic map based on SNP markers segregating in 154 progeny plants of CCxRF was constructed using JoinMap® 4.1 [32]. When markers showed an identical segregation pattern, only one was kept for the analysis. Linkage groups were made based on regression with a threshold LOD ≥ 8. Recombination frequencies were converted into map based distances in cM using Haldane’s mapping function.

Both onion-SNP markers and RF-SNPs that were polymorphic between onion and *A. roylei* were used for the production of a linkage map for the F2(CxR) population. These markers were expected to segregate in a 1:2:1 ratio. The F2 population consisted of 67 genotypes previously used for the identification and mapping of AFLP, SCAR, CAPS, and isozyme markers [20, 21] and 32 newly added F2 plants. From the original 67 genotypes, a limited set was still available (as DNA or plants) and used in the SNP analysis: 46 genotypes in the analysis with the RF-SNPs and 29 with the onion-SNPs. For mapping also marker data obtained previously were included. MapChart 2.2 [33] was used to visualize the genetic maps.

### Phenotyping for resistance to *Botrytis squamosa*

**Fig. 1:**
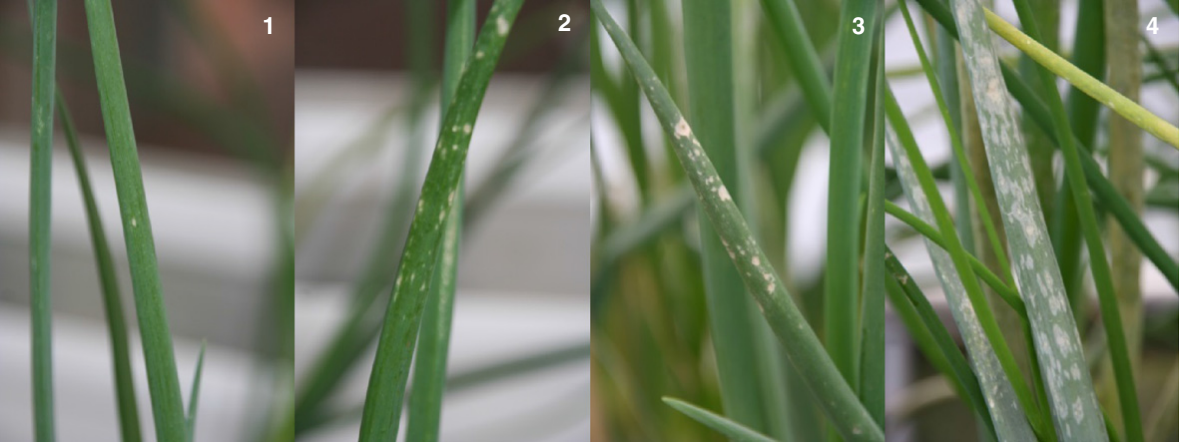
Classes of infection by *Botrytis squamosa* observed on genotypes of the CCxRF population, from left to right: Class **1** one or a few spots on a single leaf; **2** several small spots on one or two leaves; **3** large spots on one or more leaves and **4** more than 50 % of the leaves with spots

*Botrytis squamosa* isolate MUCL 31421 (Belgian Coordinated Collection of Micro-organisms, Belgium) was multiplied on water agar covered with onion leaves at 15 °C. For spore production, plates were placed under near-UV (wave length 300–400 nm) or black light (wave length 315–400 nm). Resistance tests were carried out in June 2010 (Exp. 1), September 2010 (Exp. 2) and August 2011 (Exp. 3), comprising 49, 48 and 92 genotypes (in each of the respective experiments) of the CCxRF population and one genotype each of *A. cepa* cultivar Jumbo, *A. roylei*, *A. fistulosum* and the RF hybrid. Genotypes were multiplied *in vitro* and 10 replicates per genotype were used in each experiment. The F2(CxR) population was not tested, as most plants were not available anymore. Four week old tissue-culture plants were transplanted into the greenhouse in trays containing a mixture of steamed peat soil and sand (v:v = 2:1). Two weeks later, plants were transplanted to 1.5 l pots containing onion peat soil (1 plant per pot) and kept in the greenhouse. In Exp. 1, plants were transferred 6 weeks after transplanting into a plastic fog chamber with 100 % humidity and a temperature of 15 °C (day and night) and inoculated by spraying the plants with a spore concentration of 1.10^5^ spores ml^−1^. Two days after inoculation, temperature was increased to 18 °C (day and night) and the fog chamber was removed while the humidity was kept at ~80 %. In Exp. 2 and Exp. 3, the same approach was followed but with plants of 15 and 13 weeks after transplanting, respectively. Plants were scored 3 and 4 days post inoculation (dpi) (Exp. 1), 3 and 6 dpi (Exp. 2) and 3 dpi (Exp. 3). Plants were scored in classes from 0 to 4, where 0 = no symptoms, healthy plant; 1 = a few spots on a single leaf; 2 = several small spots on more than one leaf; 3 = several larger spots on more than one leaf and 4 = more than half of the leaf covered with spots (Fig. 1).

As disease scores were obtained on an ordinal scale, the data could not be analysed under the assumption of normality. The data were modelled with reference to an underlying latent variable and threshold values associated with the ordinal scores (Proportional Odds Model) [34]. The parameters threshold values and means were estimated by the maximum likelihood method [35] employing Genstat 18th Ed. (Lawes Agricultural Trust, Rothamsted Exp. St. UK). Positions of the genotype distributions on the latent variable scale were used in subsequent QTL analyses. Broad sense heritability (H^2^) was calculated as the ratio of the genetic variation and the phenotypic variation.

### QTL mapping

The analysis of quantitative trait loci (QTLs) was performed using MapQTL® 6 [36] through interval mapping. Co-dominant markers in these regions were used as co-factors in multiple-QTL mapping (MQM). Significant LOD thresholds were determined using a genome wide permutation test with 1000 iterations. The QTL graphs were prepared with MapChart 2.2 [33].

## Results

### Transcriptome sequencing, SNP identification and development of markers

A total of 192 million RNA seq reads were obtained. The number of sequence reads varied from 5,7 million for RF to 34,6 million for Sapporo Yellow Globe. After assembly, the average contig length varied from 309 bp for ‘Bravo’ to 649 for Pukekohe Longkeeper (Table 2). The number of contigs ranged from 10,361 to 103,178. Analysis of these contigs with QualitySNP^ng^ showed that about 36 % contained reliable SNPs. For the RF genotype 7990 contigs containing a reliable SNP were identified and for the cultivars this number varied from 6709 to 38,520.

**Table 2:** General statistics of the Illumina sequencing, assembly and number of reliable SNPs identified by QualitySNP in RF and the onion cultivars. RF and cultivars Bravo and Jumbo were analyzed in a seperate run (three genotypes in one lane). In the other run two cDNA samples of eight cultivars were multiplexed per lane

Genotype	Number of bases	Number of reads	Number of contigs	N50 ^a^	Number of contigs with reliable SNPs ^b^
RF	276,285,871	5,662,574	10,361	395	7990
Bravo	572,211,306	11,727,836	46,596	309	6920
Jumbo	346,687,223	7,108,700	36,897	276	6709
Babosa	2,682,844,988	25,031,165	99,010	602	38,520
California Red	1,965,911,851	18,940,653	81,574	623	31,471
Pukekohe Longkeeper	2,274,863,215	21,244,183	99,761	649	35,203
Rio Tinto	2,017,823,470	19,525,723	81,975	588	30,996
Rumba	2,054,901,823	18,835,004	103,178	561	38,735
Sapporo Yellow Globe	3,215,089,647	34,561,122	69,206	519	27,093
South Port White Globe	2,071,923,107	29,319,916	76,187	558	28,232

^a^ Average contig length in base pairs

^b^ A SNP is called a reliable SNP when every allele is present in at least 4 reads (Tang et al. [29])

For genotyping of the CCxRF population SNPs were selected that were polymorphic in RF and monomorphic in the nine onion cultivars. Among the RF-SNPs only 2525 met this criterion (Additional file 1: Table S1). From these, 1100 (one per contig) were chosen for the development of a KASP assay (Additional file 2: Table S2).

A random selection of RF-SNPs polymorphic between onion and *A. roylei* was used for genotyping the F2(CxR) population. This set was complemented with onion-SNPs that were selected from the 243,879 onion cultivar contigs containing reliable SNPs. In 14,591 contigs 20,229 reliable onion-SNPs were identified using QualitySNP^ng^ (Additional file 3: Table S3). To maximize the chance that these SNPs would also be useful with onion cultivars, 1600 onion-SNPs were selected using the criterion that the major allele was present in at least five and the minor allele in at least four of the nine onion cultivars (Additional file 4: Table S4), and these were used for the development of KASP assays.

Functional annotations of the cDNA contigs for which KASP assays were designed were obtained using Blast2GO (www.blast2go.com) (Additional file 2: Table S2 and Additional file 4: Table S4). Successful KASP assays were designed for 767 RF-SNP markers and 1237 onion-SNP markers (success rate of 70 and 77 % respectively, see Table 3, Additional file 1: Table S1 and Additional file 3: Table S3).

**Table 3:** Numbers of SNP markers obtained with KASP assay

RF-SNPs used for development of KASP assay		1100
RF-SNP markers polymorphic in RF in KASP assay		767
polymorphic between CR	274	
polymorphic between CF	441	
not clear	52	
RF-SNP markers mapped in CCxRF		667
RF-SNP markers in F2(CxR) (all are polymorphic between CR) ^a^		119
Onion-SNPs used for development of KASP assay		1600
Onion-SNPs polymorphic between onion cultivars in KASP assay		1235
Onion-SNPs mapped in F2(CxR)		182

^*a*^ Only a selection of RF-SNP markers was used for mapping in F2(CxR)

### Molecular mapping

In total, 301 SNP markers (182 onion-SNPs, 119 RF-SNPs) were used to construct a linkage map for F2(CxR). These markers were complemented with 76 markers mapped previously [20, 21] allowing the assignment of chromosome numbers to the linkage groups. The map consisted of 805 cM divided over eight chromosomes (Additional file 5: Table S5).

For the CCxRF population 627 SNP markers segregated as expected for an AA:AB type of marker. The other 140 markers segregated in AA:AB:B0 or in A0:B0 (0 indicating a null allele) fashion when CC was A0 or 00 respectively, and were also used for mapping. The RF-SNP markers that were mapped in both F2(CxR) and CCxRF were used to assign the linkage groups of RF to chromosomes. In total 667 markers were mapped on the CCxRF map, resulting in eight linkage groups (Additional file 6: Table S6 and Additional file 7: Figure S1) with at least 35 markers per linkage group. The length of the maps varied from 83 to 207 cM, with a total length of the CCxRF map of 1051 cM. For both populations, several chromosomal regions showed skewness (with a probability < 0.001). For CCxRF, skewed regions occurred on all chromosomes, except on Chromosomes seven and eight (Additional file 5: Table S5) and for F2(CxR) such regions were seen on five of the eight chromosomes (Chromosomes 1, 3, 4, 6 and 8).

### Screening for resistance to *Botrytis squamosa*

Three independent evaluations were carried out for *B. squamosa* resistance in the CCxRF population. Leaf spots were observed in all tests already at 2 dpi. Partial resistance was observed in *A. roylei*, whereas *A. fistulosum* was almost as susceptible as onion. The level of infection of RF did not significantly differ from *A. roylei*, clearly indicating dominant inheritance of the resistance. Progeny plants of CCxRF showed a continuous variation in level of infection. Compared to the first two experiments, Exp. 3 showed the largest variation among genotypes, even though the level of infection was already high 3 dpi. In this third experiment, plants of *A. roylei* also showed infection symptoms. Heritability scores for resistance were 0.89 in Exp. 1 and Exp. 3, and in Exp. 2 0.71 (day 3) and and 0.77 (day 6).

### QTL for *B. squamosa* resistance

**Fig. 2:**
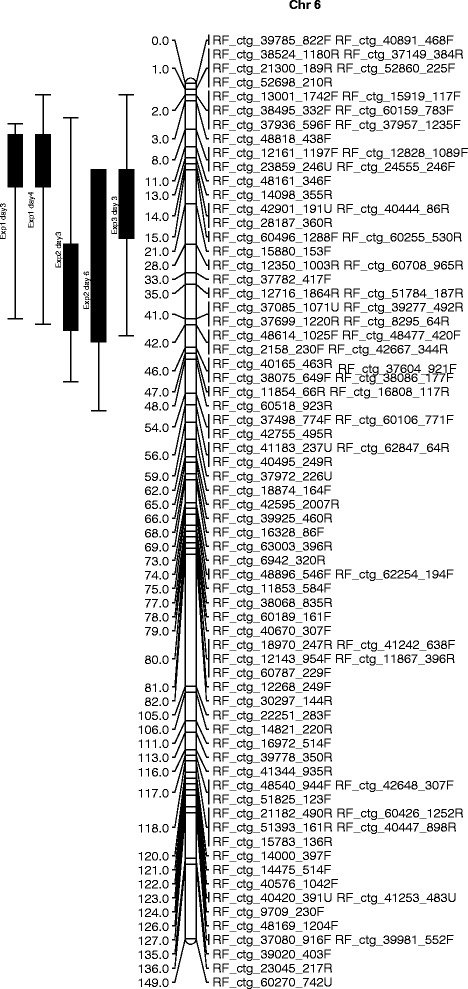
The QTL for resistance to *Botrytis squamosa* in the the CCxRF population originating from *A. roylei* identified on chromosome 6 in three independent disease tests. Lines with dashed ends show the LOD region and solid bars represent 1 LOD interval from the maximum LOD scores. Map distances are in cM

The mean values of infection over the classes as well as the mean values of infection obtained by the proportional odds model were used as input in MapQTL. For resistance to *B. squamosa*, one QTL region originating from *A. roylei* was identified on Chromosome 6 in each of the three experiments (Fig. 2). The QTL region overlapped between the three experiments, but small differences for the 1 LOD QTL interval were seen. Over the experiments, 27 to 54 % of the total variance was explained (Table 4).

**Table 4:** QTL effects from *A. roylei* for resistance to *Botrytis squamosa* in the CCxRF population identified on chromosome 6 (the highest LOD scores obtained in each test are shown)

Exp	Position	Locus	LOD	Explained variance (%)
1 day3	13	RF_ctg_14098_355R	5.31	41.2
1 day4	11	RF_ctg_48161_346F	6.86	49.7
2 day3	42	RF_ctg_48477_420F	8.01	54.4
2 day6	42	RF_ctg_48477_420F	5.20	39.9
3 day3	15	RF_ctg_60255_530R	5.91	26.6

## Discussion

### SNP discovery and marker development

SNP markers were designed to facilitate the introgression of traits from the onion related species *A. roylei* and *A. fistulosum* that possess, amongst others, disease resistances that are not present in onion cultivars. Transcriptome sequencing has proven to be an efficient approach to obtain SNP markers in diverse crops reviewed by [37, 38]. The advantage of transcriptome sequencing is that the sequencing is limited to parts of the genome that are more likely single copy, which is especially useful for crops with large, highly repetitive, genomes such as onion (16 Gb) [39–41]. We used transcriptome sequencing to obtain two sets of SNP markers that will be useful for *Allium* breeding: RF-SNP markers, which are polymorphic in the interspecific RF hybrid PRI 91021-8 and monomorphic among onion cultivars, and onion-SNP markers, which are polymorphic among onion cultivars. In total, 2525 RF-SNPs and 20,229 onion-SNPs were identified and regarded as good candidates for the development of KASP assays. Conversion of 1100 RF-SNPs and 1600 onion-SNPs in KASP genotyping assays and validation was successful for 70 % of the RF- and 77 % of the onion-SNP markers. These success rates are comparable to those reported for onion cultivars (74 %) [24] and for lily, another outcrossing species with a large genome (76 %) [30]. With the validation of 1237 new SNP markers for onion and 767 for the related species *A. roylei* and *A. fistulosum*, the amount of SNP markers that is currently publicly available has more than doubled: 43 SNP markers validated in 2005 [42], 93 in 2012 [41] and 930 in 2013 [24]. SNP markers are valuable tools for cultivar identification, determining genetic relatedness and diversity estimates among cultivars [43]. In a next step, SNP markers of various onion research groups could be combined to obtain a global consensus map of onion and other crossable *Alliaceae* [44]. Such a global consensus map is highly valuable for the onion research and breeding community, as it will allow the comparison of QTLs for specific traits detected in different populations. Mapped SNP markers are also valuable for improving the quality of the *de novo* genome assembly of onion, which is currently being carried out [45].

### Genetic maps and distortion of segregation

Selection of SNPs that are polymorphic in the RF hybrid but monomorphic in onion, turned out to be very useful for creating an inter species linkage map for *A. roylei* -*A. fistulosum*. The SNP linkage map spanned 1050 cM, which is longer than the AFLP maps of 661 cM and 886 cM earlier obtained [46, 47]. This increase in map length is likely due to the increase in the number of mapped markers at the telomeres. For the F2(CxR) population, discrepancies were encountered when mapping the newly obtained SNP markers together with 76 from the 526 previously mapped AFLP, CAPS and isozyme markers [21]. These are likely the result of the low number of identical genotypes in both studies (only 29). The intention of using the F2(CxR) population was to obtain sufficient information to assign linkage groups to chromosomes for the CCxRF population, which was successful. Distorted segregation of SNP markers was observed on six of the eight chromosomes of RF and five of the chromosomes of CR. In the past, the CCxRF population was used for the construction of a genetic linkage map based on AFLP markers [46], as well as for physical mapping [27]. Later, the population was extended with progeny plants from additional crosses between onion plants and the original RF genotype to identify QTLs for mycorrhizal responsiveness, plant dry weight and number of stem-borne roots per plant [47]. For the SNP genetic map presented in this study, progeny plants obtained from a single cross between an onion plant and the RF plant were used in order to prevent distorted segregation of markers due to unequal contributions of alleles from different mother plants. For CCxRF and F2(CxR) population, distorted segregation was reported earlier [20, 46]. Also in other crops, like tomato, distortions from the expected segregation ratio often occurs in crosses between cultivated material and wild species [48]. Distorted segregation may be the result of post- and pre-fertilization barriers between species. The phenomenon of disturbed pollen tube growth was observed in the style of interspecific F1 hybrids between *A. cepa* and *A. fistulosum* using *A. cepa* pollen [49]. Distorted segregation may also be the result of preferential genome or allele transmission, as was observed in F2 progeny plants of crosses between *A. cepa* and *A. fistulosum* as well as suppression of allelic expression and cyto-abnormalities in mitosis and meiosis [50].

### BLB resistance and mapping of the *Bs1* gene

In the current study, the focus was on the identification of the locus for resistance to *B. squamosa* from *A. roylei*. Previous studies indicated that resistance to *B. squamosa* in *A. roylei* is probably conditioned by a single dominant gene, for which *Bs1* was proposed [7]. Both *A. roylei* and the interspecific RF hybrid showed comparable low levels of infection by *B. squamosa* and were clearly resistant, whereas onion plants and plants of *A. fistulosum* were highly infected and thus susceptible. Our results also demonstrated that resistance to *B. squamosa* in *A. roylei* is based on a high level of partial resistance, indicating that plants still can be infected by the pathogen. This is in line with other studies in which Botrytis leaf blight symptoms were also observed in onion plants homozygous for the *Bs1* gene [10]. These plants were obtained after two generations of back-crossing to onion of an interspecific F1 hybrid between onion and *A. roylei*, followed by three to four generations of selfing. Under heavy disease pressure, heterozygous plants had levels of BLB symptoms between those of homozygous resistant inbred plants and susceptible controls [10]. After crossing the interspecific RF hybrid with onion, we found only one CCxRF plant with an infection level similar to *A. roylei* and RF, whereas all other plants, that we also considered resistant, had higher levels of infection. In case of one resistance gene, we would have expected a more defined segregation of resistance in this population, even if resistance was partial. Therefore, we hypothesise that one or more QTLs with minor effects originating from *A. roylei* or from *A. fistulosum* may play a role as well. Preliminary results obtained from a detached leaf assay indeed point in the direction of a minor QTL originating from *A. roylei* (Scholten and Burger, unpublished results).

QTL mapping for resistance to *B. squamosa* resulted in the identification of a QTL region on Chr. 6 of approximately 50 cM that explained 27 to 54 % of the variation over the experiments. Although this is a wide region, this is the first paper describing a locus originating from *A. roylei* conferring resistance to *B. squamosa* the causal agent of *Botrytis* leaf blight in onion. Results of earlier studies with CCxRF showed that recombination between the genomes of *A. fistulosum* and *A. roylei* and in back-crosses with onion between onion and this RF hybrid took place to a large extent [22, 23]. To narrow down the QTL region, repeated backcrossing with onion needs to be carried out followed by a recombination screening. In a recombination screening also markers that become homozygous are useful for selection.

Another approach to zoom in on the location of the *Bs1* gene is genotyping the four available BLB-resistant onion lines containing the same source of resistance, CUBLB-R1, -R2, -R3 and -R4 (generation F1BC2S3 or S4) [10]. With the use of RF-SNP markers of Chr. 6 that segregate between onion and *A. roylei* we may identify the presence of *A. roylei* fragments still present in these homozygous resistant progeny plants obtained after five or six generations of selection. Indications for applicability are favourable, as heterozygous F1 hybrids obtained after crosses between the CUBLB-R1 to -R4 lines and parental onion lines had similar levels of resistance to *B. squamosa* in most experimental field trials as the homozygous lines, without observing any drawbacks in terms of reduced bulb size or yield [10].

## Conclusions

The identification of SNP markers for onion-related species and the detection of a QTL region for resistance to *B. squamosa* described in this paper will be helpful in obtaining *B. squamosa* resistant onion cultivars for various regions in the world. Resistance to *B. squamosa* was chosen as an example, and we identified SNPs markers that will be valuable for the introgression of this and other traits from *A. roylei* and *A. fistulosum* and possibly other species into onion. Other traits may include resistance to Fusarium basal rot, pink root and *Colletotrichum.* In addition, the SNP dataset can be useful for the development of a crop that is more adapted to low levels of fertilization resulting for example from increased mycorrhizal responsiveness, or a larger root system [47].

## Additional files

Additional file 1:
**Table S1.** Sequences flanking reliable single nucleotide polymorphisms (SNPs) in RF cDNA RF_ctgs conducive for genotyping using the KASP platform. (XLSX 244 kb) Additional file 2:
**Table S2.** Results of Blast2Go to obtain functional anotation of RF contigs containing SNPs that were selected for the KASP platform. (XLSX 57 kb) Additional file 3:
**Table S3.** Sequences flanking reliable single nucleotide polymorphisms (SNPs) in onion cDNA al_contigs conducive for genotyping using the KASP platform. (XLSX 1413 kb) Additional file 4:
**Table S4.** Results of Blast2Go to obtain functional anotation of onion contigs containing SNPs that were selected for the KASP platform. (XLSX 101 kb) Additional file 5:
**Table S5.** Positions and Chi-square values to expected segregation ratios for SNPs markers segregating in the F2(CxR) population. (XLSX 42 kb) Additional file 6:
**Table S6.** Positions and Chi-square values to expected segregation ratios for SNPs markers segregating in the CCxRF_new population based on RF-SNP markers. (XLSX 57 kb) Additional file 7:
**Figure S1.** Aligned genetic maps of the CCxRF (left) and the F2(CxR) populations (right). (DOCX 201 kb)

## Abbreviations

AFLP: Amplified fragment length polymorphism
BLB: Botrytis leaf blight
CAPS: Cleaved amplified polymorphic sequence
CCxRF: The interspecific three way cross between *A. cepa* and the F1 hybrid between *A. roylei* and *A. fistulosum*
dpi: days post inoculation
Exp.: Experiment
F2(CxR): F2 of onion x *A. roylei*
onion-SNPs: SNPs polymorphic among onion cultivars
QTL: Quantitative Trait Locus
RF SNPs: SNPs that are polymorphic in RF and monomorphic in onion cultivars
RF: The interspecific F1 hybrid between *A. roylei* and *A. fistulosum*
SCAR: Sequence characterized amplified region
SNP: Single nucleotide polymorphism
