# Supplementary material for: SNP-markers in Allium species to facilitate introgression breeding in onion
Source: BMC Plant Biol. 2016 Aug 31;16(1):187. doi: 10.1186/s12870-016-0879-0 (PMC5006257; doi:10.1186/s12870-016-0879-0)
Supplement: Additional file 7: — Figure S1. Aligned genetic maps of the CCxRF (left) and the F2(CxR) populations (right). (DOCX 201 kb) [file 12870_2016_879_MOESM7_ESM.docx]

Supplemental Fig. 1 Aligned genetic maps of the CCxRF (left) and the F2(CxR) populations (right). RF-SNP markers (names start with RF_ctg ) are markers that are polymorphic between *A. roylei* and *A. fistulosum*. These markers were mapped in both populations. In the F2(CxR) also onion-SNP markers (names start with al_ctg) were mapped, these are markers that are polymorphic between onion cultivars and between onion and *A. roylei*. Previously mapped markers, such as AFLP, SCAR, CAPS and isozyme markers are also included in the F2(CxR) population [21,22]. The red lines show the alignments between both maps.
